# Targeting immunoglobulin superfamily member 9 (IGSF9) to overcome acute myeloid leukemia resistance to CAR-T therapy

**DOI:** 10.1186/s13046-026-03740-4

**Published:** 2026-05-19

**Authors:** Xianhui Meng, Fangmin Li, Hong Yu, Chunling Li, Yuxiao Sun, Hongying Wang, Guantong Liu, Jiashen Zhang, Lijun Hui, Fang Li, Shuping Wei, Yaopeng Wang, Zunling Li

**Affiliations:** 1 Shandong Key Lab of Complex Medical Intelligence and Aging, Shandong Medicine and Health Key Lab of Respiratory Infection and Tumor Immunity, Department of Biochemistry and Molecular Biology, Shandong Tumour Immunotherapy Research Innovation Team, Shandong Medical And Pharmaceutical University, Yantai, Shandong 264003 P.R. China; 2 Department of Thoracic Surgery, The First Affiliated Hospital of Shantou University Medical College, Shantou University Medical College, Shantou, Guangdong 515041 P.R. China; 3 Department of Thoracic Surgery, Qingdao Hospital, University of Health and Rehabilitation Sciences (Qingdao Municipal Hospital), Qingdao, Shandong 266011 P.R. China

**Keywords:** AML, CAR-T, IGSF9, Immune suppression

## Abstract

**Background:**

CAR-T cell therapy faces substantial barriers in AML, yet the mechanisms by which AML evades CAR-T cell cytotoxicity—through tumor-intrinsic factors and microenvironmental suppression—remain poorly defined. IGSF9 has recently been identified as an immunosuppressive molecule selectively expressed on AML blasts, but its role in mediating resistance to CAR-T therapy is unknown.

**Methods:**

We examined IGSF9 expression in AML cells upon CAR-T challenge and conducted in vitro and in vivo assays to define its functional impact on CAR-T cell activity. To overcome IGSF9-mediated suppression, we evaluated two therapeutic strategies: antibody-mediated IGSF9 blockade and the generation of IGSF9-specific CAR-T cells (IG9BBz).

**Results:**

CAR-T–induced cytotoxic pressure upregulated IGSF9 on AML cells. IGSF9-positive AML cells exhibited resistance to CAR-T killing and impaired CAR-T persistence both in vitro and in vivo. Antibody blockade of IGSF9 restored CAR-T function in xenograft models. Moreover, IG9BBz CAR-T cells demonstrated potent and selective elimination of IGSF9-positive AML cells in both settings.

**Conclusions:**

Our findings identify a cytokine-driven IGSF9 resistance circuit that suppresses CAR-T cell function in AML. Therapeutic disruption of this pathway through IGSF9 blockade or IGSF9-specific CAR-T cells restores antitumor immunity and provides complementary strategies to overcome CAR-T resistance in AML.

**Supplementary Information:**

The online version contains supplementary material available at 10.1186/s13046-026-03740-4.

## Background

AML is an aggressive hematologic malignancy characterized by clonal expansion of myeloid progenitors. It accounts for over 80% of adult acute leukemias [1]. Despite advances in therapy, the prognosis remains poor, with 5-year survival rates stagnating at 20–30%. Although FLT3 inhibitors and venetoclax-based regimens have improved outcomes for certain patient subgroups, elderly and high-risk cohorts experience relapse rates exceeding 60%. Allogeneic hematopoietic stem cell transplantation, though potentially curative, faces significant limitations including donor scarcity, GVHD and relapse rates greater than 30% [2–4]. These unmet clinical needs underscore the imperative for novel therapeutic strategies in AML.

CAR-T cell therapy, which enables MHC-independent tumor targeting, has revolutionized the treatment of B-cell malignancies, achieving remission rates exceeding 90% in relapsed/refractory disease [5]. However, its translation to AML has been largely ineffective, with clinical response rates consistently below 30% despite promising preclinical targets such as CD33, CD123, CLL1 [6–8]. This therapeutic failure stems not only from antigenic heterogeneity but also from the profoundly immunosuppressive AML microenvironment [9–12]. The mechanisms by which AML evades CAR-T cell cytotoxicity-through both intrinsic tumor factors and microenvironmental suppression-remain poorly understood.

IGSF9 is a transmembrane protein composed of five immunoglobulin (Ig)-like domains, two fibronectin type III (FNIII) domains, a transmembrane region and an intracellular immunoreceptor tyrosine-based inhibitory motif (ITIM) [13]. Originally implicated in neurodevelopment, IGSF9 is aberrantly overexpressed in multiple carcinomas, where it correlates with poor prognosis and drives metastasis through the induction of epithelial-to-mesenchymal transition [14–17]. Critically, we recently identified IGSF9 as a pan-cancer immunosuppressive checkpoint that orchestrates immune-evasive microenvironments by directly impairing cytotoxic lymphocyte function. Blockade of IGSF9 reversed T cell dysfunction and suppressed tumor growth in various tumor models [18]. Notably, we observed highly restricted IGSF9 expression on leukemic blasts from AML patients, with minimal or absent expression in normal hematopoietic stem cells and most other major tissues [19]. This tumor-selective expression profiles IGSF9 as a compelling antigenic target for AML immunotherapy.

IFNγ has been identified as a major inducer of IGSF9 expression in AML [19]. Given that IFNγ is abundantly secreted by activated CAR-T cells, we hypothesized that cytokine-driven upregulation of IGSF9 contributes to AML resistance against CAR-T therapy. However, the mechanisms by which AML cells exploit IGSF9 to evade immune surveillance, particularly CAR-T–mediated cytotoxicity, remain undefined. Here, we delineate the role of IGSF9 in orchestrating AML immune escape and engineer next-generation IG9BBz CAR-T cells to neutralize this immunosuppressive pathway. Our findings establish a mechanistic and therapeutic framework for overcoming resistance in AML immunotherapy.

## Methods

### Cells and patient samples

All cell lines were preserved by our lab and verified by STR [18, 19]. The AML cell lines (THP-1, MOLM13, U937, MV4-11) were cultured in RPMI1640 medium (10% FBS, 1% penicillin/streptomycin), The adherent cells (293T, LL/2, A549, H1299) were cultured in DMEM medium (10% FBS, 1% penicillin/streptomycin). Human primary T cells were cultured in RPMI1640 medium (10% FBS, 1% penicillin/streptomycin, 5ng/ml IL-7, 5ng/ml IL-15). Mouse primary T cells were cultured in RPMI1640 medium (10% FBS, 1% penicillin/streptomycin, 10ng/ml IL-2). Fresh bone marrow aspirates of AML patients were obtained from Yantai Yuhuangding Hospital with informed consent.

### Production of CAR-T cells

Human PBMC were isolated from healthy blood, which obtained from Yantai central blood station. Then the cells were stimulated by CD3/CD28 Dynabeads (ThermoFisher, Cat.No.11141D) with a ratio of 1:1 and cultured with RPMI1640 medium (10% FBS, 1% PS, 5ng/ml IL-7, 5ng/ml IL-15). To transduce CAR genes, the lentivirus was produced in 293T cells which has been transfected with CAR plasmid and packing plasmids (MD2.G and PsPAXP2). Transduction was performed 48 h after PBMC isolation. The activated T cells were transduced with lentivirus supernatants on RetroNectin (Takara, Cat.No.T100A) coated plates by centrifuge at 2,000×g for 2 h at 32℃. Transduction efficiency of CAR-T cells was determined after three days.

To generate mouse CAR-T cells, the splenocytes were isolated from C57BL/6 wild type mice and activated by Concanavalin A (2 µg/ml). To prepare retrovirus for transduction, the GP2-293 cells were transfected with CAR plasmid and pCAG-Eco envelop vector. 48 h after transfection, the retrovirus supernatants were collected and concentrated by centrifuge at 24,000×g for 2 h at 4℃. Transduction of CAR-T cells were performed 24 h after splenocytes isolation on RetroNectin coated plats by spin centrifugation.

### Co-culture of CAR-T cells with AML cells

MOLM13-CD19 or MOLM13-CD19-IGSF9 cells (5 × 10⁵) were co-cultured with 19BBz or 33BBz CAR-T cells (5 × 10⁵) in 24-well plates using complete RPMI 1640 medium. After 20 h, cells were harvested for flow cytometry. PD-L1 and IGSF9 surface expression was evaluated on gated CD19⁺ MOLM13 cells. To assess IGSF9 on antigen-negative AML cells, MOLM13-CD19 and MOLM13-GFP cells were mixed 1:1. This mixture (5 × 10⁵ cells) was co-cultured with an equal number of CAR-T cells under identical conditions. For conditioned medium experiments, 33BBz CAR-T cells and MOLM13 cells were co-cultured (1:1 ratio). After 20 h, medium was collected, filtered (0.45 μm), and mixed 1:1 with fresh complete RPMI 1640. MOLM13, U937, MV4-11 cells or patient-derived AML cells were then treated with this mixture for 24 h, followed by IGSF9 surface expression analysis via flow cytometry. For IGSF9 induction by IFNγ, MOLM13 cells were treated with 100ng/ml IFNγ (MCE, Cat.No.HY-P7025) for 24 h. For IFNγ blockage, 20 ng/mL Emapalumab (MCE, Cat.No.HY-P99191) was added to the conditioned medium prior to treatment of MOLM13 cells for 24 h. To assess degranulation (CD107a) and cytokines production, 1 × 10⁵ 33BBz CAR-T cells were co-cultured with equal numbers of MOLM13 or MOLM13-IGSF9 cells in complete RPMI1640 medium supplemented with 3 µg/ml Brefeldin A (MCE, Cat.No.HY-16592) and 1mM Monensin (MCE, Cat.No.HY-N0150) in a 96-well plate, then incubated for 6 h prior to flow cytometry analysis.

### Repeated stimulation of CAR-T cells

For repeated stimulation, irradiated THP-1 (WT) or THP-1 (IGSF9 KO) cells (5 × 10⁵) were seeded in 24-well plates. CD33-specific CAR-T cells (5 × 10⁵) were added (E/T = 1/1). Fresh target cells were replenished at the same E/T ratio every 3 days. On day 12 following four stimulations, CAR-T cells were harvested and differentiation states were assessed by flow cytometry.

### C57BL/6 mouse model

Four- to six-week-old female C57BL/6 mice were purchased from Charles River Laboratories. Individual mice were randomly assigned to treatment/control groups Mice (*n* = 4/group) received subcutaneous injections of 1 × 10⁶ LL/2-hCD19 or LL/2-hCD19-mIGSF9 cells. When tumors reached ~ 50 mm³ (day 9), 2 × 10⁶ 19m28z CAR-T cells were administered intravenously. Mice were euthanized two weeks post-CAR-T infusion. Tumors were enzymatically dissociated, and exhaustion/differentiation states of tumor-infiltrating GFP⁺ CAR-T cells were assessed by flow cytometry.

### Xenograft mouse model

Four- to six-week-old female NOD/SCID-IL2Rg-null (NSG) mice were purchased from Shanghai Model Organisms. Individual mice were randomly assigned to treatment/control groups. For xenograft model with IGSF9 blockade, NSG mice received intravenous injection of 0.5 × 10⁶ luciferase-expressing, GFP labeled MOLM13-IGSF9 cells. Three days post engraftment, mice were administered 0.5 × 10⁶ 33BBz CAR-T or UnT cells via tail vein. Anti-IGSF9 blocking antibody or mouse IgG control (0.2 mg/mouse) was delivered intravenously starting 24 h post CAR-T infusion, with repeat dosing every 4 days (total 3 doses). Tumor burden was quantified weekly by bioluminescence imaging (IVIS Spectrum, PerkinElmer). For flow cytometric analysis of CAR-T cell function, mice received two antibody doses every 3 days beginning 24 h post CAR-T infusion. At the terminal endpoint (day 12 post CAR-T infusion), the peripheral blood, bone marrow and spleen tissue were harvested. The frequency of GFP⁺ tumor cells and mCherry⁺ CAR-T cells in vivo was determined by flow cytometry. CAR-T cell differentiation status was evaluated based on surface expression of CD45RA and CD62L.

For in vivo experiments of IGSF9-specific CAR-T cells, NSG mice received intravenous injection of 0.2 × 10⁶ luciferase-expressing MOLM13-IGSF9 cells. Three days post engraftment, mice were administered 2 × 10⁶ IG9BBz CAR-T or UnT cells via tail vein. Tumor burden was quantified weekly by bioluminescence imaging. Mice survival was monitored until death or reaching humane endpoint (Severe weight loss or paralysis). The probability of survival was described by Kaplan-Meier curves using log-rank (Mantel-Cox) tests. To quantify tumor burden and adoptive T cell persistence, bone marrow was harvested from mice at 14 days post-T cell infusion. GFP⁺ tumor cells and infused T cells were subsequently analyzed by flow cytometry to determine their frequency and phenotype.

For IGSF9 endogenously expressed AML model, NSG mice were engrafted with THP-1 tumors via intravenous injection of 0.5 × 10⁶ THP-1-GFP-Luc cells. The next day, 5 × 10⁶ IG9BBz CAR-T cells or UnT cells were administered by tail vein injection. Tumor burden was monitored longitudinally through serial bioluminescence imaging. Bone marrow was harvested at day 25 post-T cell infusion, followed by flow cytometric analysis of GFP⁺ tumor cells and infused T cells.

### Flow cytometry

The production of human IGSF9 antibody was described previously [18]. To detect IGSF9 by flow cytometry, cells were incubated with IGSF9 antibody for 30 min at room temperature, and then stained with APC-conjugated anti-mouse IgG antibody (Biolegend, Cat.No.405308) for 20 min at 4℃ in the dark. Direct staining of surface antigens was performed by incubating cells with fluorochrome-conjugated antibodies for 20 min at 4℃ in the dark. For intracellular staining of IFNγ and TNFα, cells were fixed and permeabilized using a Fixation/Permeabilization buffer (eBioscience, Cat.No.00-5123-43). Antibodies targeting human PD-L1 (Cat.No.329734), CD19 (Cat.No.302208), CD3 (Cat.No.300317), CD4 (Cat.No.317432), CD8 (Cat.No.344750), CD107a (Cat.No.328609), TNFα (Cat.No.376203), IFNγ (Cat.No.502510) PD-1 (Cat.No.367406), TIM-3 (Cat.No.345041), LAG-3 (Cat.No.369317), CD45RA (Cat.No.304142), CD62L (Cat.No.304810), CD69 (Cat.No.310904) and mouse PD-1 (Cat.No.135221), TIM-3(Cat.No.134008), CD44 (Cat.No.103012), CD62L (Cat.No.104424), CD13 (Cat.No.301703), CD33 (Cat.No.303441), CD117 (Cat.No.313217) were purchased from Biolegend. Flow cytometry was performed on a BD Fortessa flow cytometer (BD Bioscience) or an Aurora Spectral Flow Cytometer (Cytek). Data were analyzed with FlowJo software (V10, TreeStar).

### Western blot

A549 and A549-IGSF9-HA cells were lysed using RIPA buffer (Solarbio) containing protease inhibitors (Beyotime Biotechnology). The samples were first loaded onto an SDS-PAGE gel and then transferred to a PVDF membrane. The following primary antibodies were used for protein probing: mouse anti-human IGSF9(1:1000 dilution, home-made), mouse anti-HA tag (1:6000 dilution, Proteintech, Cat.No.51064-2-AP), Rabbit anti-human GAPDH (1:6000 dilution, Affinity, Cat.No.AF7021). After incubation with HRP-conjugated secondary antibodies (Proteintech) and ECL reagent (Biosharp), the protein bands were visualized by a chemiluminescent imaging system (Tanon).

### In vitro cytotoxicity assay

CAR-T cell cytotoxicity was assessed using a luciferase-based assay. Target cells expressing luciferase (5 × 10⁵) were co-cultured with CAR-T cells at specified E: T ratios in 96-well black-bottom plates. After 18–20 h, D-luciferin substrate was added, and bioluminescence (BL) was measured using a luminescence reader (LUX-P110, BLT). Lysis rates were calculated as [(1- BL of each sample)/BL of target cells alone] ×100%.

### IGSF9-ECD-Fc binding assay

Jurkat T cells expressing 19BBz or IG9BBz CAR constructs (1 × 10^6^) were incubated with 2 µg IGSF9-ECD-Fc protein (prepared as described [18]) or human IgG1 control at 4℃ for 30 min. Cells were washed with PBS, then stained with APC-conjugated anti-human IgG Fc antibody (Biolegend, 410712) at 4℃ for 30 min.

### Cytokines quantification by ELISA

IG9BBz CAR-T cells were co-cultured with K562 or K562-IGSF9 cells at 1:1 ratio in complete RPMI1640 medium in a six-well plate. After 20 h, culture supernatants were collected. Human IFNγ ELISA Kit (Abcam, ab46025), Human TNFα SimpleStep ELISA Kit (Abcam, ab181421) and Human IL-2 ELISA Kit (Proteintech, KE00017) were used to quantify IFNγ, TNFα and IL-2 secretion following manufacturer’s instruction.

### Bioinformatic analysis of IGSF9 expression in AML

TCGA-LAML RNA-seq data (*n* = 173) and normal hematopoietic cell data from GTEx (*n* = 1,048) were downloaded and analyzed for IGSF9 expression using R (version 4.4.1). An independent AML cohort from cBioPortal (Daniel et al., Cancer Cell 2022; *n* = 649) was analyzed for IGSF9 expression across myeloid malignancies and correlated with clinical parameters by using cBioPortal online tools. Overall survival analysis was performed using Kaplan-Meier method with log-rank test in R.

### Statistical analysis

GraphPad Prism 8 was used for statistical analysis. A two-tail Student’s t-test was used for comparing two groups. Data are presented as mean ± SEM. *P < 0.05, **p < 0.01, ***p < 0.001, ****p < 0.0001.

## Results

### AML with high IGSF9 expression correlates with poor prognosis

**Fig. 1:**
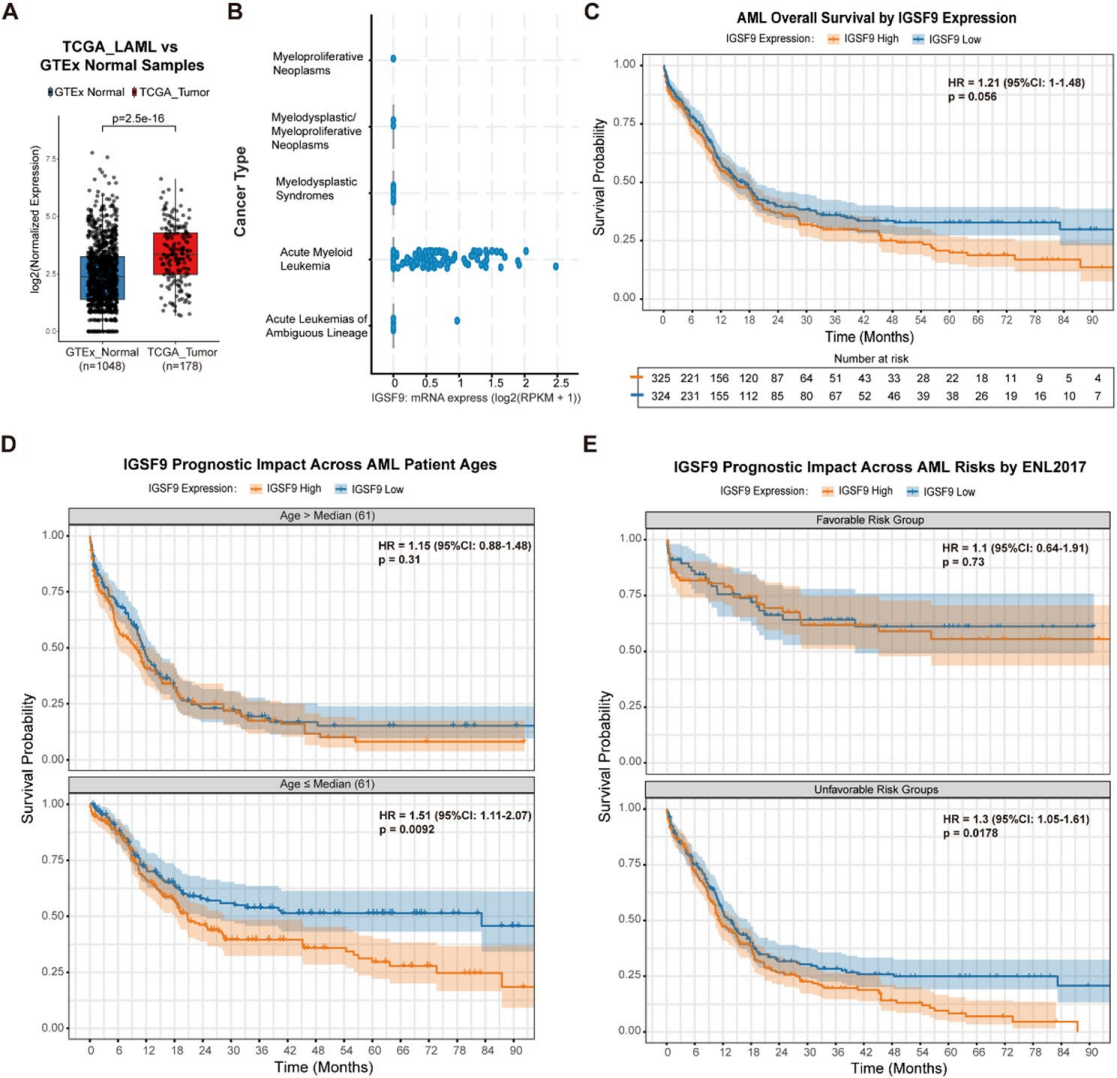
Clinical correlation of IGSF9 in AML. **A** Comparative analysis of IGSF9 expression in TCGA-LAML samples (n = 178) versus normal hematopoietic cells from GTEx (n = 1048). **B** IGSF9 Expression profiling across myeloid malignancies in the cBioPortal cohort (Daniel et al., Cancer Cell 2022). **C** Overall survival by IGSF9 expression in total AML cohort. **D** Overall survival by IGSF9 expression in younger (age ≤ 61) and older (age > 61) AML patients. **E** Overall survival by IGSF9 expression across ENL2017 risk subgroups

To comprehensively address the clinical relevance of IGSF9 in AML, we analyzed two clinical cohorts of AML cases. Initial comparison of TCGA-LAML data (*n* = 173) versus normal hematopoietic cells (*n* = 1048) confirmed significant IGSF9 upregulation in AML (Fig. 1A). Given the limited clinical annotation in TCGA, we extended our analysis to an AML cohort from cBioPortal (*n* = 649) [20, 21]. In this cohort, IGSF9 showed preferential enrichment in AML compared to other myeloid malignancies (Fig. 1B). IGSF9 expression was not remarkably associated to disease stage, FAB subtypes or CEBPA mutation status (Supplementary Figure S1). Survival analysis demonstrated a trend toward inferior outcomes in the full cohort (HR = 1.21, 95% CI: 0.99–1.48; *p* = 0.056; Fig. 1C). Notably, this association achieved statistical significance in younger patients (Age ≤ 61 years; HR = 1.51, *p* = 0.0092) (Fig. 1D) and ELN2017 unfavorable-risk patients (HR = 1.30, *p* = 0.0178) (Fig. 1E). These findings establish IGSF9 as an independent prognostic marker identifying AML patients with aggressive disease biology.

### CAR-T cell cytotoxicity induces IGSF9 upregulation in AML cells

**Fig. 2:**
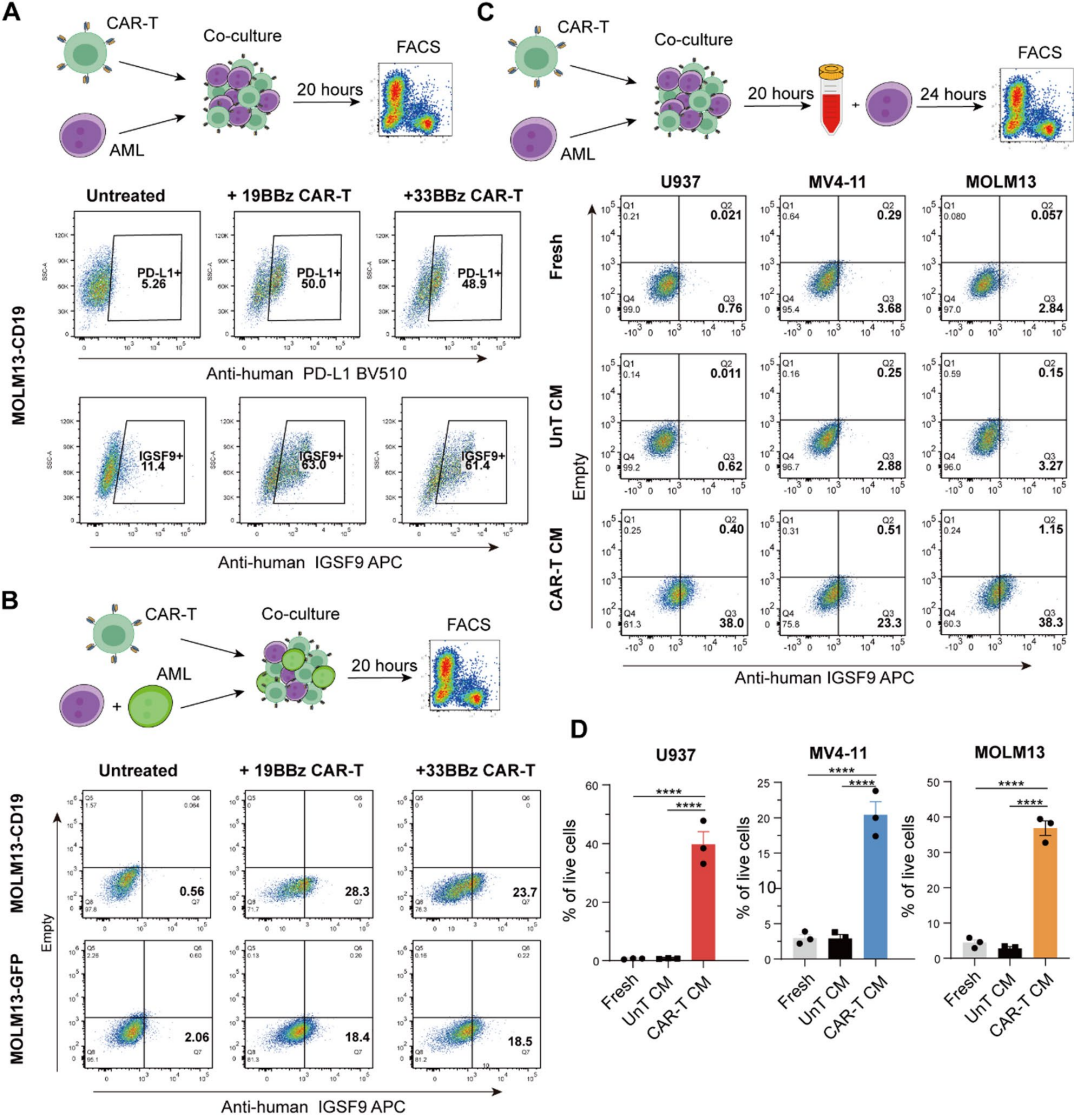
CAR-T cell co-culture induces contact-independent IGSF9 upregulation in AML cells. **A** IGSF9 and PD-L1 expression on MOLM13-CD19 cells after co-cultured with 19BBz or 33BBz CAR-T cells for 20 h (n = 1). **B** IGSF9 upregulation in antigen-expressing (CD19⁺GFP⁻) and bystander (CD19⁻GFP⁺) AML cells after CAR-T co-culture (n = 1). **C** Conditioned medium from MOLM13/33BBz CAR-T co-culture induces IGSF9 upregulation in AML cell lines (n = 3). **D** Quantification of IGSF9 expression proportion from panel C (mean ± SEM; n = 3)

Given that IGSF9 is an IFNγ-inducible immune checkpoint molecule and CAR-T cells release cytotoxic cytokines (e.g., IFNγ) upon tumor engagement [19], we hypothesized that CAR-T cell attack triggers IGSF9 expression on AML cells. Co-culturing AML cells with second-generation 4-1BBz CAR-T cells targeting CD33 (33BBz; CD33 as an endogenous AML antigen) or CD19 (19BBz; CD19 as a clinically validated target) significantly upregulated both PD-L1 (confirming IFNγ pathway activation) and IGSF9 on target MOLM13-CD19⁺ cells after 20 h (Fig. 2A, Supplementary Figure S2A). Mechanistically, in mixed co-cultures containing both MOLM13-CD19⁺ and MOLM13-GFP⁺ cells, both populations exhibited comparable IGSF9 upregulation when exposed to either 19BBz or 33BBz CAR-T cells, confirming antigen-independence induction (Fig. 2B, Supplementary Figure S2B). Furthermore, conditioned medium from 33BBz CAR-T/MOLM13 co-cultures, unlike that from UnT cell controls, robustly induced IGSF9 expression in fresh AML cells (Fig. 2C, D, Supplementary Figure S2C). Exogenous IFNγ recapitulated this effect (Supplementary Figure. S2D). The inducible response mediated by CAR-T conditioned medium was completely abrogated by IFNγ blockade with Emapalumab (Supplementary Figure. S2E), establishing IFNγ as the principal driver. These findings demonstrate that soluble mediators especially IFNγ, released during CAR-T cells/tumor interactions drive IGSF9 upregulation in AML cells, thereby establishing a feedforward inhibitory loop.

### IGSF9 expression on tumor cells impairs CAR-T cell anti-tumor functions

**Fig. 3:**
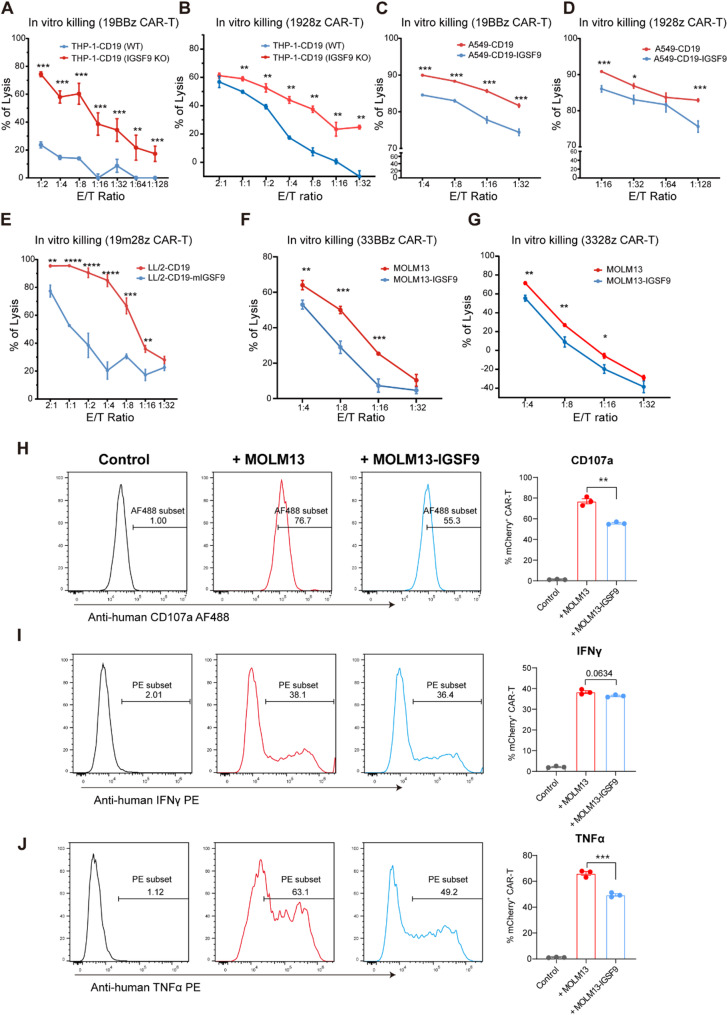
IGSF9 expression on tumor cells impairs CAR-T cell cytotoxic function. **A**–**G** CAR-T cell cytotoxicity against luciferase-expressing tumor cells was assessed by luminescence after 18–20 h of co-culture. **A** Cytotoxicity of 19BBz CAR-T cells against THP-1-CD19 versus IGSF9-knockout (KO) THP-1-CD19 cells. **B** Cytotoxicity of 1928z CAR-T cells against THP-1-CD19 versus IGSF9-knockout (KO) THP-1-CD19 cells. **C** Cytotoxicity of 19BBz CAR-T cells against A549-CD19 versus A549-CD19 overexpressing IGSF9 (A549-CD19-IGSF9). **D** Cytotoxicity of 1928z CAR-T cells against A549-CD19 versus A549-CD19-IGSF9. **E** Cytotoxicity of mouse 19m28z CAR-T cells against LL/2-CD19 versus LL/2-CD19 overexpressing mouse IGSF9 (LL/2-CD19-mIGSF9). **F** Cytotoxicity of 33BBz CAR-T cells against MOLM13 versus MOLM13 overexpressing IGSF9 (MOLM13-IGSF9). **G** Cytotoxicity of 3328z CAR-T cells against MOLM13 versus MOLM13 overexpressing IGSF9 (MOLM13-IGSF9). **H** Surface expression of CD107a on mCherry+ 33BBz CAR-T cells after six hours co-culture with MOLM13 or MOLM13-IGSF9 cells (1:1 ratio) (mean ± SEM; n = 3). **I** IFNγ^+^ frequency of mCherry^+^ 33BBz CAR-T cells post-co-culture (mean ± SEM; n = 3). **J** TNFα^+^ frequency of mCherry^+^ 33BBz CAR-T cells post-co-culture (mean ± SEM; n = 3)

**Fig. 4:**
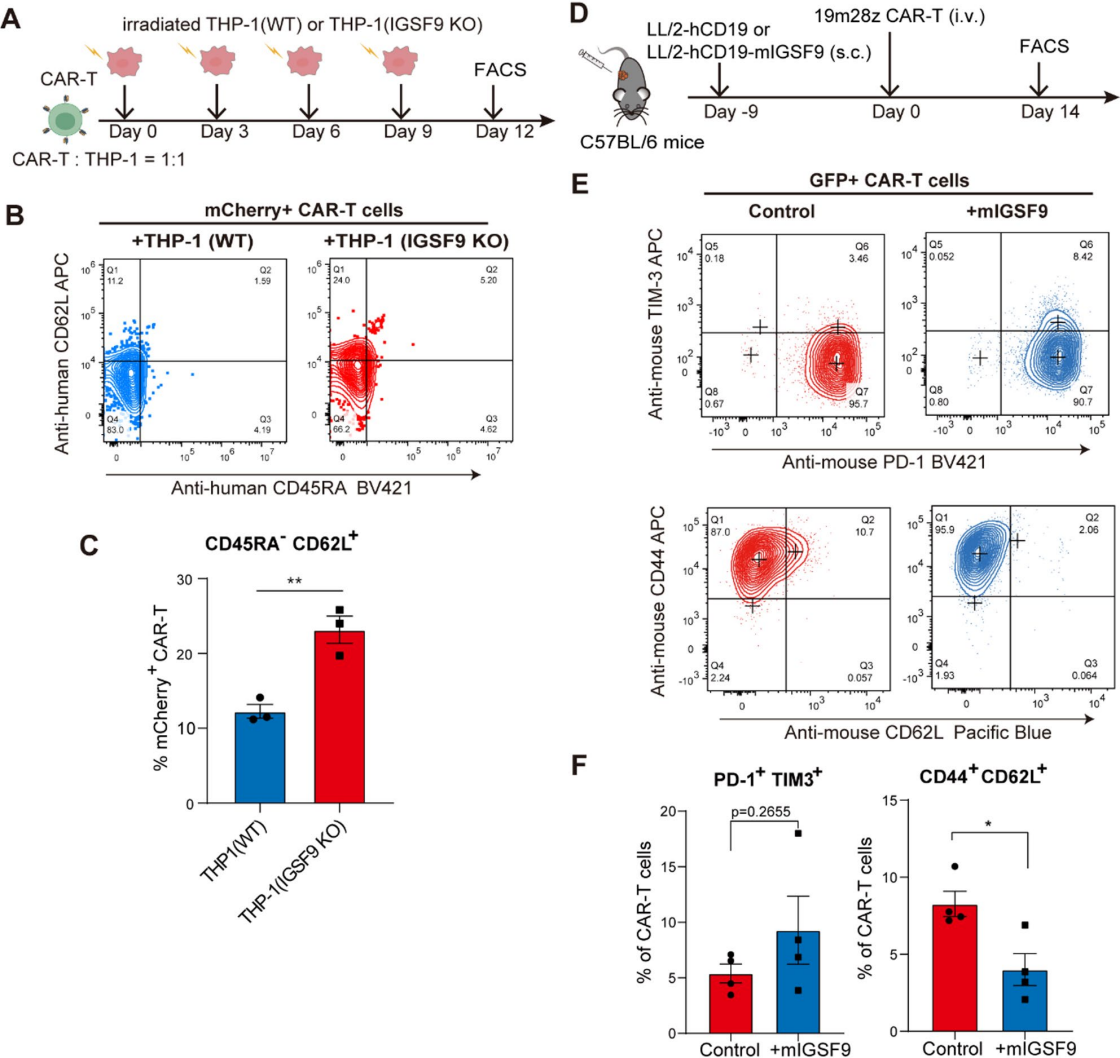
IGSF9 expression on tumor cells impaired CAR-T cells persistence. **A** Treatment schema: CD33-specific CAR-T cells were repeatedly stimulated with irradiated THP-1 (WT) or THP-1 (IGSF9 KO) cells at a ratio of 1:1 for four rounds (three days per round). **B** Representative dot plots of CD45RA and CD62L expression on mCherry+ CAR-T cells. **C** Quantification of central memory (CD45RA^−^CD62L^+^) CAR-T cells from (B) (mean ± SEM; n = 3). **D** Treatment schema: C57BL/6 mice engrafted with LL/2-CD19 or LL/2-CD19-mIGSF9 tumors were treated with GFP^+^ mouse 19m28z CAR-T cells. After two weeks, CAR-T cells were collected and analyzed by flow cytometry. **E** Representative dot plots of PD-1 vs. TIM-3 (exhaustion) and CD44 vs. CD62L (differentiation) on tumor-infiltrating GFP^+^ CAR-T cells. **F** Quantification of PD-1^+^TIM-3^+^ and CD44^+^CD62L^+^ CAR-T cells from (E) (mean ± SEM; n = 4)

To determine whether tumor-expressed IGSF9 suppresses CAR-T cell activity, we performed luciferase-based cytotoxicity assays using CAR-T cells with different constructs (Supplementary Figure S3A). Various CAR-T target cells were generated to express CD19 antigen and different level of IGSF9 (Supplementary Figure S3B). Knockout of IGSF9 in THP-1-CD19⁺ cells significantly enhanced tumor lysis by both 19BBz and 1928z CAR-T cells (Fig. 3A, B). Conversely, IGSF9 overexpression in A549-CD19⁺ cells markedly reduced killing by both 19BBz and 1928z CAR-T cells (Fig. 3C, D), indicating suppression independent CAR structure. Murine-derived 19m28z CAR-T cells targeting LL/2 cells in which human CD19 (hCD19) was overexpressed exhibited significantly diminished cytotoxicity against LL/2-hCD19-mIGSF9⁺ cells compared to LL/2-hCD19 controls, confirming similar suppressive function of mouse-derived IGSF9 (Fig. 3E). Similarly, MOLM13-IGSF9⁺ cells showed enhanced resistance to 33BBz and 3328z CAR-T cell-mediated killing compared to parental MOLM13 (Fig. 3F, G). As a control, 1928z CAR-T cells targeting MOLM13 or MOLM13-IGSF9^+^ cells did not elicit remarkable tumor lysis effect (Supplementary Figure S3C). Mechanistically, co-cultured with MOLM13-IGSF9 elicited lower expression of CD107a of 33BBz CAR-T cells than MOLM13 groups (Fig. 3H), indicating repressed degranulation of CAR-T cells by IGSF9. The cytokines release was also compared. CAR-T cells stimulated by MOLM13-IGSF9 produced comparable level of IFNγ but significantly reduced level of TNFα compared to MOLM13 groups (Fig. 3I, J). Moreover, repeated stimulation of CD33-specific CAR-T cells with irradiated THP-1 (IGSF9 KO) cells (vs. THP-1 WT) elevated the frequency of CD45RA^−^CD62L^+^ central memory T cells (Fig. 4A-C). In vivo, tumors derived from LL/2-hCD19-mIGSF9⁺ cells in CAR-T-treated mice exhibited reduced CD44⁺CD62L⁺ memory T cell subsets and increased (though not significant, *p* = 0.2655) PD-1⁺TIM-3⁺ population compared to LL/2-hCD19⁺ controls (Fig. 4D-F).

Collectively, these data demonstrate that tumor-intrinsic IGSF9 impairs CAR-T cell functions by suppressing T cells degranulation, cytokines release and memory differentiation.

### Blockade of IGSF9 enhances CAR-T cell anti-tumor activity in vivo

**Fig. 5:**
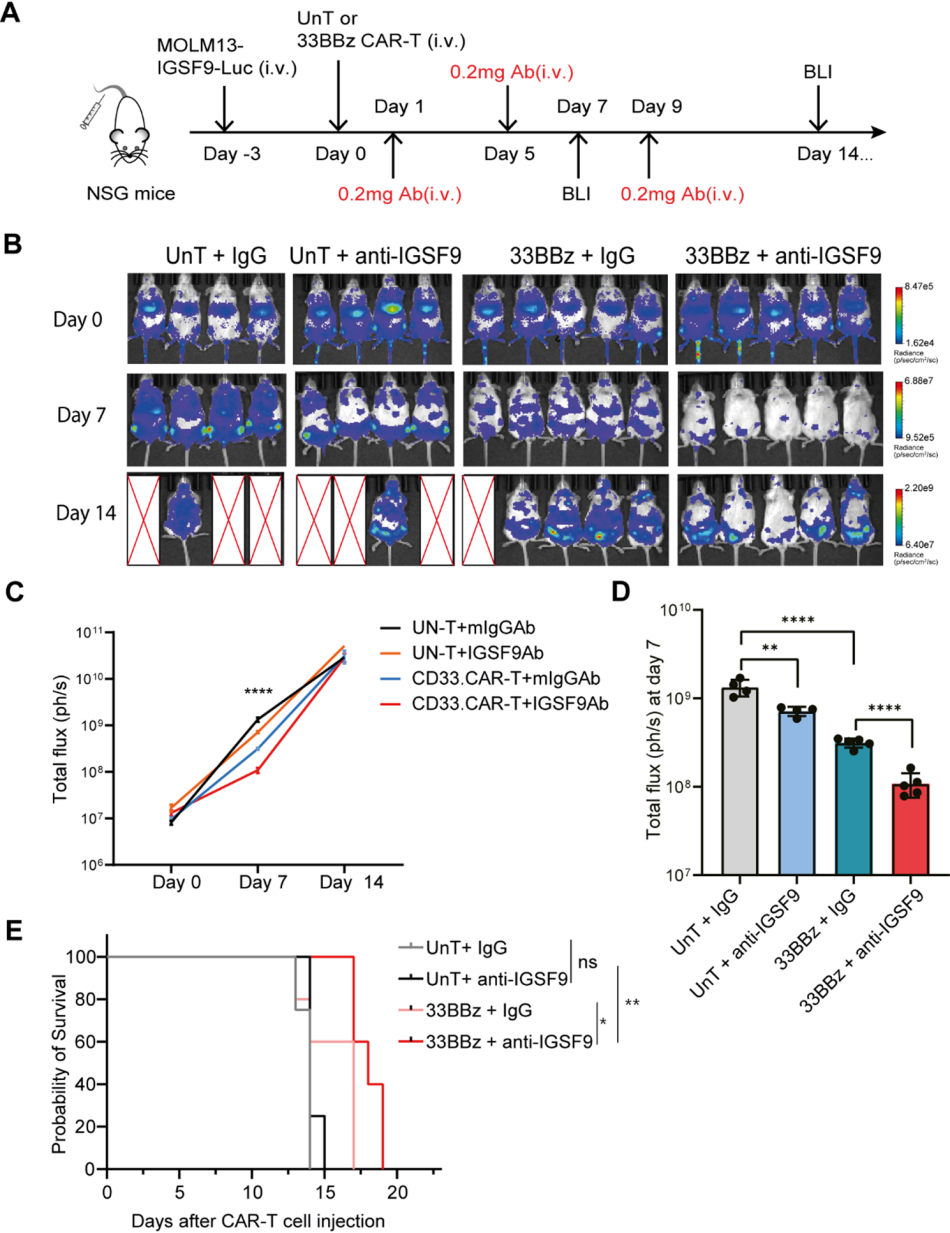
Combined IGSF9 blockade synergizes with CAR-T cell therapy to control tumor growth. **A** Treatment schema: Mice engrafted with MOLM13-IGSF9-GFP cells received 0.5 × 10⁶ 33BBz CAR-T cells or UnT, followed by three doses of anti-IGSF9 or IgG control antibody (0.2 mg/dose) (n = 4 per group for UnT; n = 5 per group for CAR-T). **B** Representative bioluminescence images showing tumor burden dynamics. **C** Quantification of total flux (photons/second) across experiment (mean ± SEM, n = 4 per group for UnT; n = 5 per group for CAR-T). **D** Statistical assay of the total flux at day 7 (mean ± SEM, n = 4 per group for UnT; n = 5 per group for CAR-T). **E** Kaplan-Meier survival curves (n = 4 per group for UnT; n = 5 per group for CAR-T)

While IGSF9 blockade has been shown to improve T cell function in solid tumors [18], its potential to enhance CAR-T cell efficacy against AML in vivo remained unexplored. To address this, we employed a combination therapy approach in an AML xenograft model. NSG mice engrafted with GFP labeled MOLM13-IGSF9 cells were administered either 33BBz CAR-T cells or UnT. Starting one day after CAR-T cells infusion, mice were administered anti-IGSF9 or isotype control IgG every four days for a total of three doses (Fig. 5A). Bioluminescence imaging revealed a significant reduction in tumor burden by day 7 in CAR-T cell-treated mice compared to UnT controls. Notably, anti-IGSF9 co-administration further suppressed tumor progression in the CAR-T group (Fig. 5B-D). Furthermore, CAR-T cell treatment significantly prolonged survival, and this effect that was further enhanced by anti-IGSF9 treatment (Fig. 5E).

**Fig. 6:**
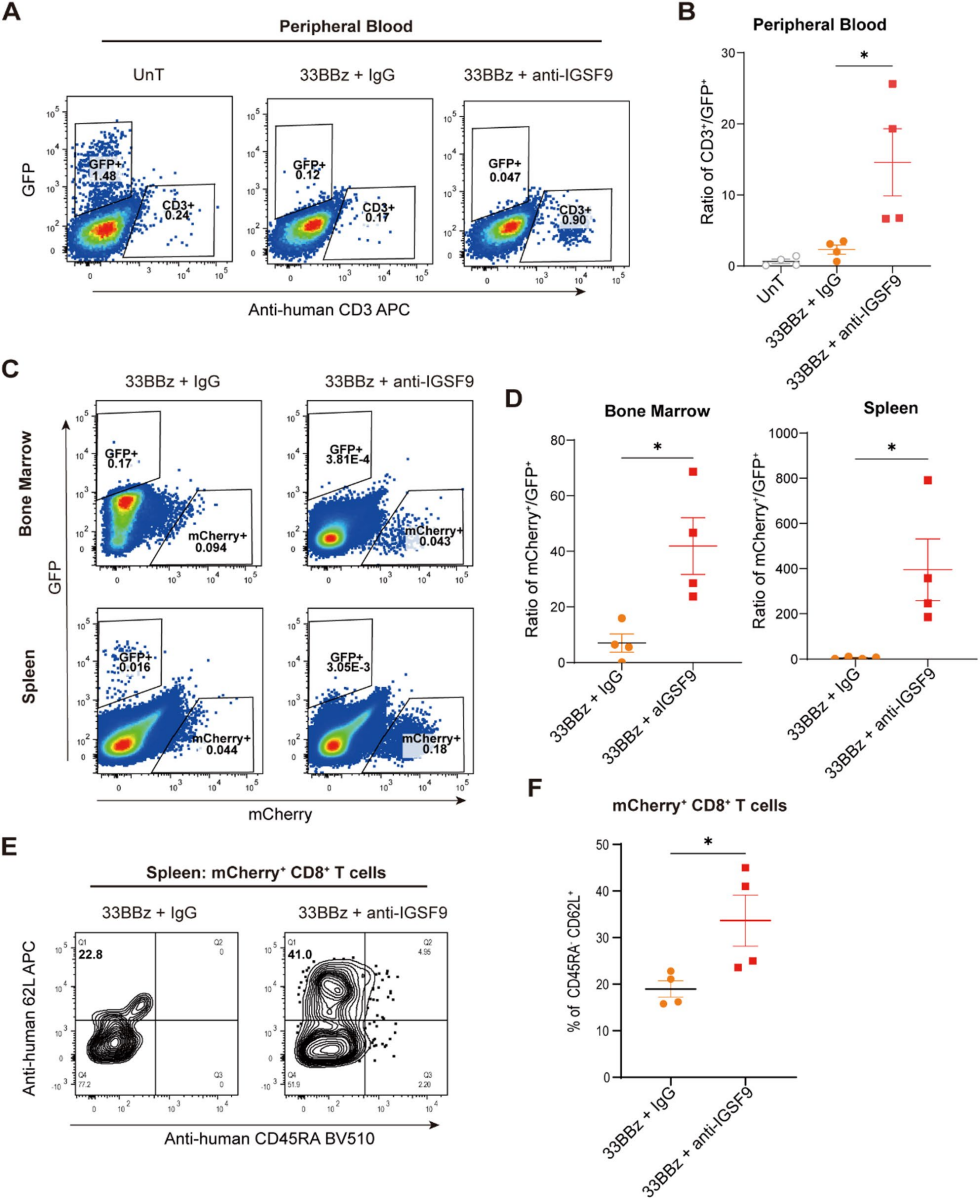
IGSF9 blockade enhances CAR-T cell persistence and memory differentiation in tissues. **A** Representative dot plots of tumor burden (GFP^+^ cells) and infused T cell (human CD3^+^) frequency in peripheral blood 12 days post-infusion (after two antibody doses). **B** Quantification of tumor and CAR-T cell frequencies in peripheral blood (mean ± SEM; n = 4 mice/group). **C** Tumor burden and CAR-T cell (mCherry^+^) frequency in bone marrow and spleen. **D** Quantification of tumor and CAR-T cell frequencies in bone marrow/spleen (mean ± SEM; n = 4 mice/group). **E** Central memory differentiation (CD45RA^−^CD62L^+^) of splenic CD8^+^CAR-T cells (mCherry^+^). **F** Quantification of central memory CD8^+^CAR-T cells frequencies in spleen (mean ± SEM; n = 4 mice/group)

We next evaluated the impact of anti-IGSF9 treatment on the persistence of CAR-T cells in vivo. One day after CAR-T cell injection, tumor-engrafted mice received anti-IGSF9 or control IgG, administered in two doses every four days. Flow cytometry analysis at 12 days post-CAR-T injection revealed that 33BBz CAR-T cells significantly reduced the frequency of GFP⁺ tumor cells (Fig. 6A, Supplementary Figure S4A). Notably, in anti-IGSF9 combination group, GFP^+^ tumor cells were nearly undetectable (Fig. 6A). Furthermore, anti-IGSF9 treatment markedly increased the proportion of human CD3⁺ T cells in peripheral blood (Fig. 6A, B). We then quantified CAR-T cells (mCherry⁺) in the bone marrow and spleen. Compared to IgG controls, anti-IGSF9 treatment elevated the proportion of mCherry⁺ CAR-T cells in these organs, resulting in a significantly higher mCherry⁺/GFP⁺ cell ratio (Fig. 6C, D). Finally, we evaluated the differentiation state of CAR-T cells in the spleen, and found that anti-IGSF9 treatment significantly increased the proportion of CAR-T cells exhibiting a central memory phenotype (CD45RA⁻CD62L⁺) (Fig. 6E, F, Supplementary Figure S4B).

Collectively, these results suggest that IGSF9 blockage enhances CAR-T cells persistence and promotes CAR-T cells anti-AML functions in vivo.

### Design and functional validation of IGSF9-specific CAR-T cells

**Fig. 7:**
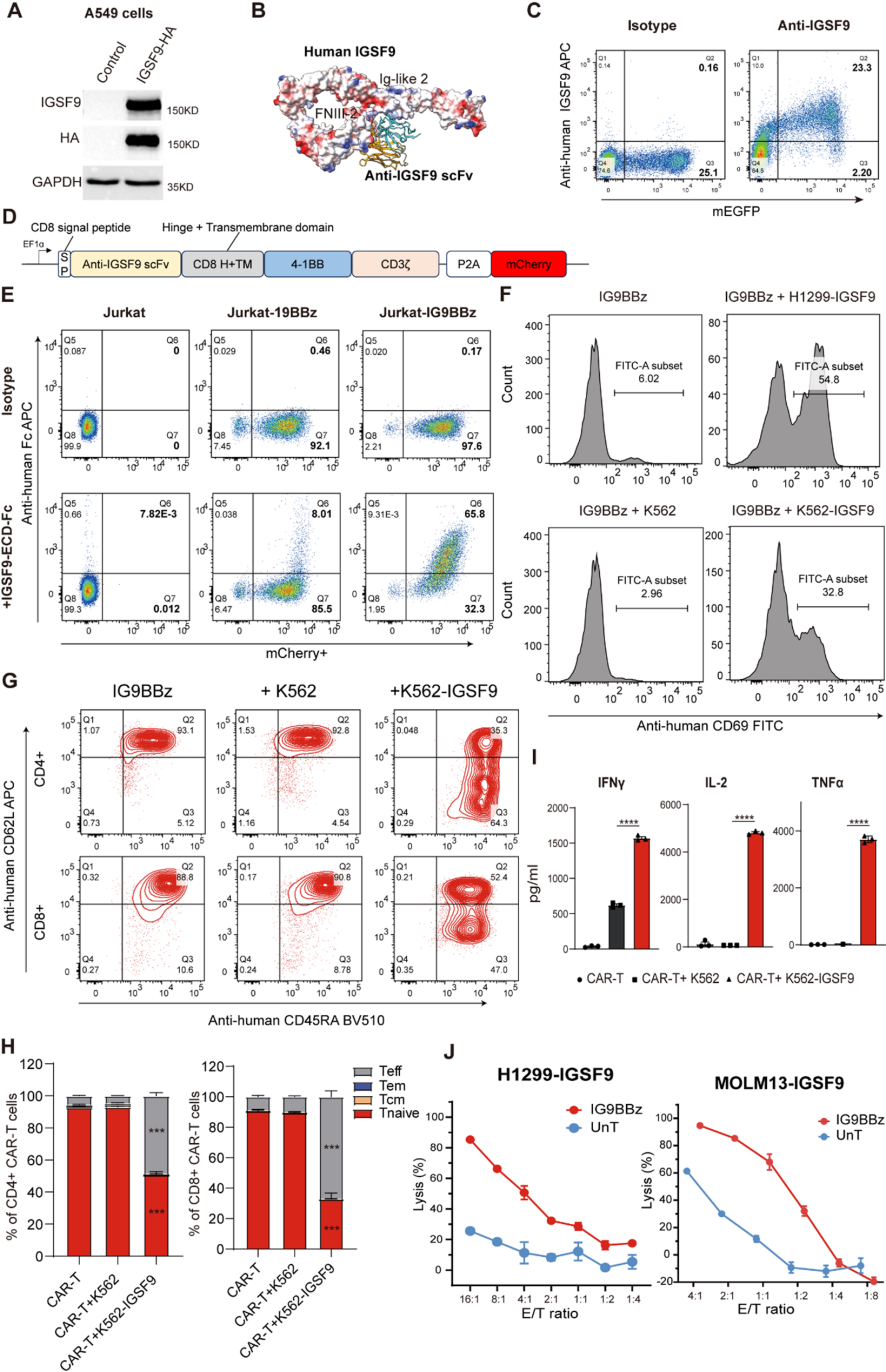
Antigen specificity of IGSF9-specific CAR construct. **A** anti-IGSF9 antibody recognized IGSF9-HA overexpression in engineered A549-IGSF9-HA cells versus parental A549 controls by western blot. **B** Epitope binding of anti-IGSF9 scFv to human IGSF9 ECD as predicted by Alphafold3. **C** anti-IGSF9 antibody recognized IGSF9-mEGFP surface expression in transiently transfected 293T cells by flow cytometry. **D** IG9BBz CAR architecture. The CAR contains an anti-IGSF9 scFv, CD8 hinge and transmembrane region, 41BB co-stimulatory domain and CD3ζ signaling domain. A mCherry reporter was linked downstream of CAR via a P2A self-cleaving peptide. CAR expression was driven by the EF1α promoter. **E** Specific binding of IGSF9-ECD-Fc fusion protein to IG9BBz CAR^+^ Jurkat cells versus control (19BBz CAR). **F** Representative histograms of CD69 expression on IG9BBz CAR-T cells after 20-hour co-culture with IGSF9+ tumor lines (H1299-IGSF9, K562-IGSF9) versus IGSF9− controls (K562) at 1:1 E: T ratio. **G** Memory phenotype of CAR-T cells using CD45RA and CD62L post-co-culture with K562-IGSF9. **H** Memory subset distribution in CD4^+^ and CD8^+^ CAR-T cell compartments (mean ± SEM; n = 3; subsets defined: Tcm [CD45RA^−^CD62L^+^], Tem [CD45RA^−^CD62L^−^], Tnaive [CD45RA^+^CD62L^+^], Teff [CD45RA^+^CD62L^−^]). **I** Antigen-dependent cytokine secretion (IFNγ, IL-2, TNFα) measured by ELISA following 20-hour co-culture with K562-IGSF9 versus parental K562 (mean ± SEM; n = 3). **J** Dose-dependent cytotoxicity of CAR-T cells against IGSF9^+^ solid (H1299-IGSF9) and liquid (MOLM13-IGSF9) tumor models at indicated E: T ratios (mean ± SEM; n = 3)

Given the restricted expression of IGSF9 to AML blasts and its role as a critical suppressor of T-cell function, we hypothesized that selective elimination of IGSF9⁺ AML cells would limit disease progression. To this end, we engineered novel IGSF9-specific CAR-T cells (IG9BBz). Western blot analysis confirmed the specificity of the anti-IGSF9 antibody for IGSF9⁺ human cells (Fig. 7A). Structural modeling predicted that the Ig-like 2 and FNIII-2 domains constitute the core epitope recognized by the scFv antibody (Fig. 7B), which is consistent with our previous report [19]. Flow cytometry demonstrated robust labeling of membrane-expressed IGSF9-mEGFP in 293T cells using the fluorophore-conjugated anti-IGSF9 antibody, confirming efficient binding to cell-surface IGSF9 (Fig. 7C). The IG9BBz CAR construct incorporated an scFv derived from the anti-IGSF9 antibody as the antigen-binding domain, coupled with CD8 hinge and transmembrane region, 4-1BB and CD3z intracellular signaling domains, and a P2A-linked mCherry reporter for tracking CAR expression (Fig. 7D). Jurkat cells expressing IG9BBz bound recombinant IGSF9-ECD-Fc, whereas control CAR variants lacking the scFv showed no binding, confirming antigen specificity (Fig. 7E). To evaluate antigen-dependent activation, primary IG9BBz CAR-T cells were generated and co-cultured with tumor targets for 20 h (Supplementary Figure S5). Exposure to H1299-IGSF9 or K562-IGSF9 cells, but not parental K562 cells, markedly upregulated CD69 expression on CAR-T cells (Fig. 7F). Moreover, co-culture with K562-IGSF9 cells, but not parental K562 cells, induced a pronounced shift of CAR-T cells from a naïve to an effector state (Fig. 7G, H), indicating antigen-specific activation of IG9BBz CAR-T cells. To further determine CAR-T cell functions, cytokine secretion was quantified by ELISA. Co-culture with K562-IGSF9 cells, but not parental K562 cells elicited abundant secretion of IFNγ, IL-2, and TNFα (Fig. 7I). In cytotoxicity assays, IG9BBz CAR-T cells efficiently eliminated H1299-IGSF9 and MOLM13-IGSF9 cells within 18 h, as measured by bioluminescence (Figure. 7 J). In tumor cells overexpressing IGSF9 and CD19, IG9BBz CAR-T cells exhibited cytotoxicity comparable to that of 19BBz CAR-T cells (Supplementary Figure S5D).

### Potent anti-AML activity of IG9BBz CAR-T cells across preclinical models

**Fig. 8:**
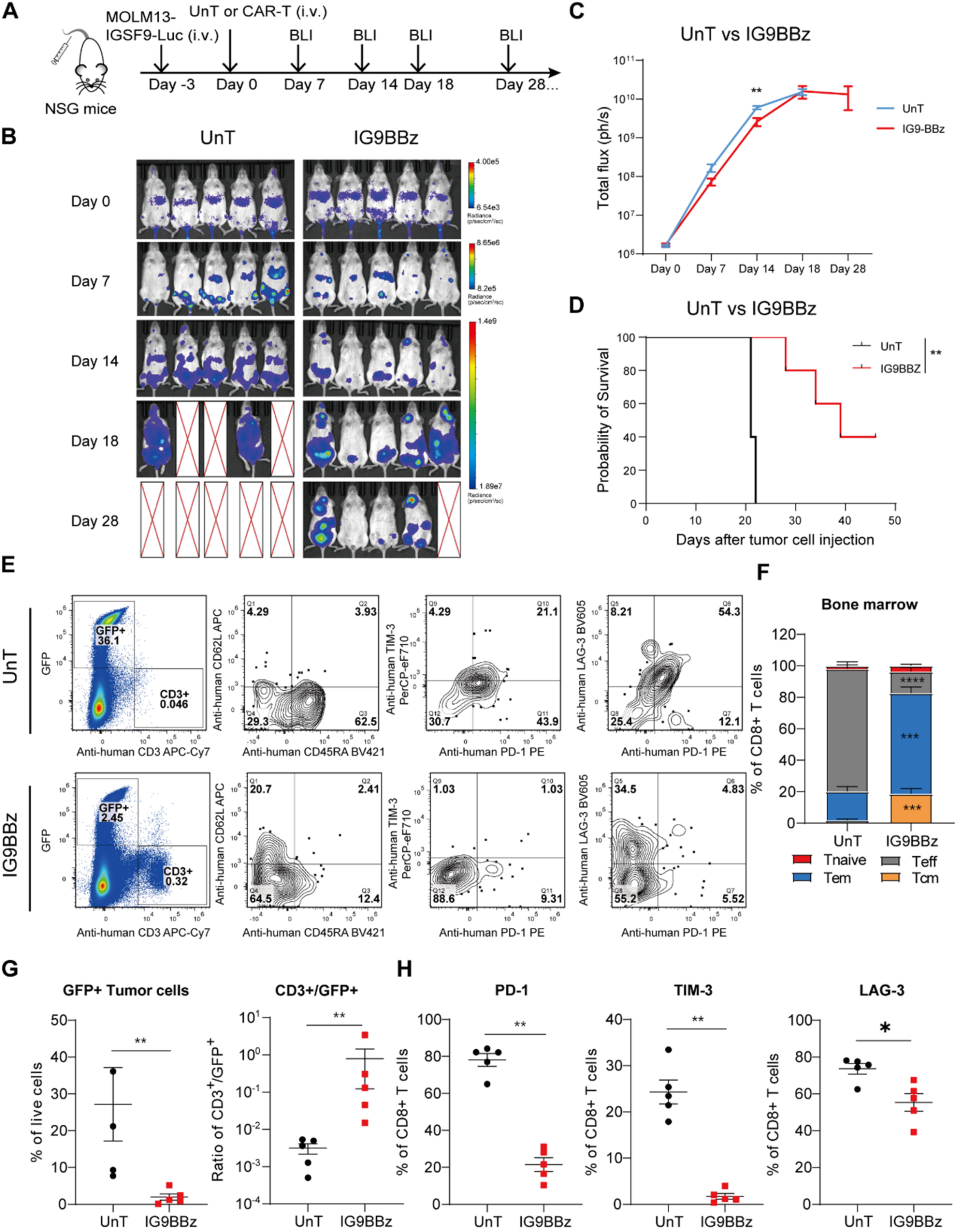
IG9BBz CAR-T cells eliminate IGSF9^+^ MOLM13 xenografts and confer survival advantage. **A** Treatment schema: NSG mice (n = 5 per group) received intravenous injection of 0.2 × 10⁶ MOLM13-IGSF9-GFP cells, followed by 2 × 10⁶ IG9BBz CAR-T cells or UnT three days later. **B** Bioluminescence images showing tumor burden reduction in CAR-T-treated mice at serial timepoints. **C** Quantitative tumor burden (total flux, p/s; mean ± SEM). **D** Significant survival extension in CAR-T-treated cohort (Kaplan-Meier curves by log-rank test). **E** Representative flow cytometry plots of the tumor/T cell frequencies, T cells differentiation and exhaustion in bone marrow. **F **Memory subset distribution in UnT and mCherry^+^ CAR-T cell compartments (mean ± SEM; *n* = 5). **G** Quantification of GFP^+^ tumor frequencies and CD3^+^/GFP+ ratio (mean ± SEM; n = 5). **H** Quantification of T cell inhibitory receptors (PD-1, TIM-3 and LAG-3) (mean ± SEM; n = 5)

The anti-tumor efficacy of IG9BBz CAR-T cells was first evaluated in NSG mice bearing MOLM13-IGSF9-Luc AML xenografts (Fig. 8A). IG9BBz CAR-T cell administration significantly reduced tumor burden, as quantified by bioluminescence imaging (Fig. 8B, C), and extended survival compared to UnT controls (Fig. 8D). To confirm direct tumor elimination, bone marrow was analyzed at 14 days post-infusion. IG9BBz CAR-T treatment markedly decreased GFP⁺ tumor burden while increasing the CD3⁺/GFP⁺ ratio (Figs. 8E, G). Residual tumor cells maintained IGSF9 expression in both groups (Supplementary Figure S6A). Notably, the IGSF9% in residual GFP⁺ cells were significantly lower in IG9BBz-treated mice versus UnT controls, indicating preferential targeting of IGSF9-high tumor cells (Supplementary Figure S6B). Phenotypic characterization revealed distinct T-cell differentiation patterns: UnT cells predominantly exhibited an effector phenotype (CD45RA⁺CD62L⁻), whereas IG9BBz CAR-T cells (CD3⁺mCherry⁺) primarily comprised effector memory (CD45RA⁻CD62L⁻) and central memory (CD45RA⁻CD62L⁺) subsets (Figs. 8E, F). Critically, IG9BBz CAR-T cells demonstrated significantly lower expression of inhibitory receptors (PD-1, TIM-3, LAG-3) versus UnT controls (Figs. 8E, H). These findings indicate tumor antigen-driven activation and favorable differentiation of IG9BBz CAR-T cells. The emergence of memory subpopulations coupled with reduced exhaustion suggests enhanced persistence potential of this CAR construct.

**Fig. 9:**
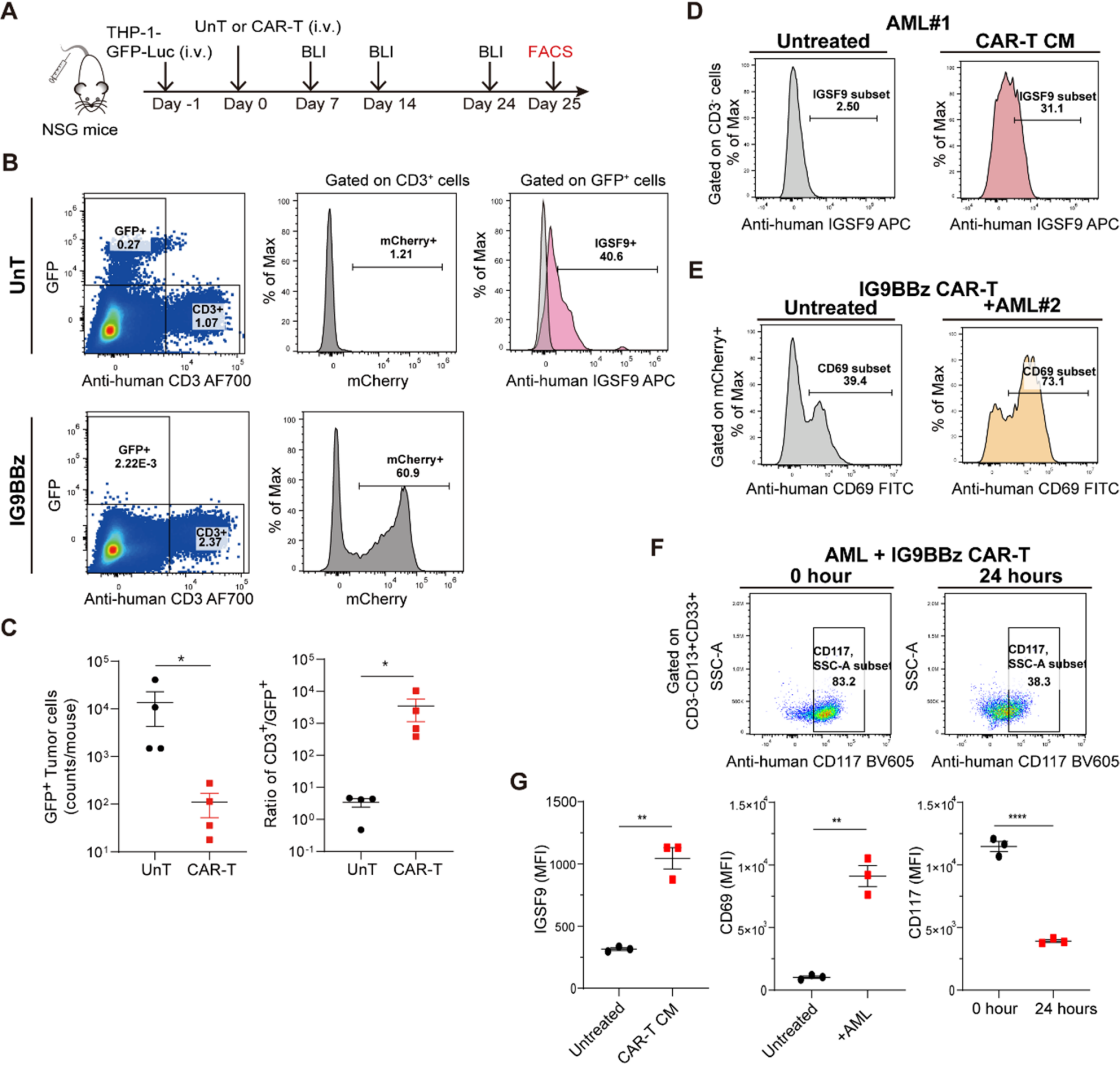
Anti-tumor effect of IG9BBz CAR-T Cells on AML cells with endogenous IGSF9 expression. **A** Treatment schema: NSG mice (n = 4 per group) received intravenous 0.5 × 10⁶ THP-1-GFP-Luc cells followed by 5 × 10⁶ IG9BBz CAR-T cells or UnT at day + 1. **B** Representative bone marrow analysis: GFP^+^ tumor infiltration vs. adoptive T cell engraftment. **C** Quantified GFP^+^ tumor cells and CD3^+^/GFP^+^ ratios in bone marrow (mean ± SEM; n = 4). **D** Representative IGSF9 expression of patient-derived AML cells (PD-AML) treated with conditioned medium from MOLM13/33BBz CAR-T co-cultures (CAR-T CM) (mean ± SEM; n = 3). **E** CD69 activation on IG9BBz CAR-T cells after 24 h co-culture with PD-AML (mean ± SEM; n = 3). **F** Ratio of CD3^−^ CD13^+^CD33^+^CD117^+^ AML blasts before and after IG9BBz CAR-T cells treatment. **G** Quantification of the flow cytometry analysis from (D) to (F) (mean ± SEM; n = 3)

To evaluate IG9BBz CAR-T cell activity against AML with endogenous IGSF9 expression, NSG mice bearing THP-1-GFP-Luc xenografts received IG9BBz CAR-T cells or UnT controls (Fig. 9A). Longitudinal bioluminescence imaging revealed progressive tumor expansion in UnT-treated mice, whereas 3 of 4 IG9BBz CAR-T-treated mice exhibited minimal disease progression (Supplementary Figure S6C). Bone marrow analysis at day 25 post-infusion confirmed significant tumor reduction by IG9BBz CAR-T treatment, demonstrating decreased GFP⁺ tumor burden and increased CD3⁺/GFP⁺ ratios (Fig. 9B, C). The GFP^+^ tumor cells were further verified to be IGSF9 positive (Fig. 9C). Notably, one IG9BBz-treated mouse showing elevated bioluminescent signal exhibited low marrow tumor infiltration, suggesting predominant extramedullary tumor engraftment in this individual.

To assess clinical relevance, bone marrow aspirates from two AML patients were analyzed. Treatment with conditioned medium from 33BBz CAR-T/MOLM13 co-cultures significantly upregulated IGSF9 expression in the CD3⁻ compartment of AML (Fig. 9D, G). When co-cultured with AML blasts, IG9BBz CAR-T cells exhibited elevated CD69 expression, confirming antigen-specific activation (Fig. 9E, G). Notably, IGSF9⁺ cells predominantly resided within the primitive CD13⁺CD33⁺CD117⁺ population (Supplementary Figure S6D). IG9BBz CAR-T co-culture selectively reduced this target-enriched subset in AML samples (Fig. 9F, G).

Collectively, these data establish that IG9BBz CAR-T cells exert potent anti-leukemic activity against IGSF9-expressing AML in vivo, and demonstrate translational potential through CAR-T-induced IGSF9 upregulation that enhances blast elimination.

## Discussion

Immune escape remains a major barrier to durable remission after CAR-T therapy for AML. Here, we identify IGSF9 as an immune suppressor that drives resistance to CAR-T cells in AML. Cytotoxic cytokines released by activated CAR-T cells induced robust IGSF9 expression on AML blasts. The release of inflammatory cytokines, including IFNγ, IL-2, and TNFα, represents a primary mechanism by which CAR-T cells exert cytotoxic effects [22]. Our previous work identified IFNγ as a key driver of IGSF9 upregulation via the JAK1-STAT1 signaling pathway [19]. Whether additional cytokines contribute to this induction remains to be determined. Nevertheless, we show that the cytokine milieu generated during CAR-T cytotoxicity is sufficient to induce substantial IGSF9 expression in AML cells. This antigen-independent induction by soluble mediators establishes a feed-forward immunosuppressive loop: CAR-T cells activation triggers cytokine release, which in turn upregulates IGSF9 on AML blasts, thereby amplifying its inhibitory effect and progressively impairing T-cell function. This mechanism represents a distinct form of immune evasion that is independent of antigen loss [23]. Disrupting this immune-evasive niche is paramount for advancing AML CAR-T therapy.

 In vitro, AML cells with high IGSF9 expression confer treatment resistance against CAR-T cytotoxicity, potentially through impairing effector functions—specifically by suppressing degranulation and TNFα secretion. Repeated antigen stimulation further compromised CAR-T memory differentiation, thereby undermining durable antitumor immunity. Although the structural basis of IGSF9 signaling remains undefined, two non-mutually exclusive mechanisms are plausible: (1) direct activation of inhibitory pathways, dampening CAR-T cell activation, and (2) signaling through the IGSF9 intracellular ITIM motif in AML cells, potentially enhancing their survival upon T cell contact. The identity of the cognate IGSF9 receptor remains challenging. Definitive validation of IGSF9-interacted molecules warrants further investigation.

Using xenogeneic IGSF9 positive AML models, we demonstrate that monoclonal antibody–mediated IGSF9 blockade attenuates tumor progression and prolongs survival. Beyond direct blockade of IGSF9’s inhibitory signaling, the modest tumor control observed with anti-IGSF9 plus UnT cells points to an additional contribution from antibody-dependent cellular phagocytosis (ADCP). Nevertheless, In vivo, IGSF9 inhibition enhanced CAR-T cell persistence, restored the CD62L⁺CD44⁺ central-memory T cell pool, and reduced the frequency of exhausted T cell subsets. These findings establish IGSF9 as a tractable therapeutic target capable of reprogramming the AML microenvironment from a tolerogenic to an immunogenic state.

We further developed IGSF9-specific CAR-T cells designed to integrate tumor antigen recognition with simultaneous ablation of an inhibitory ligand. IGSF9 expression is restricted to leukemic blasts and absent on normal CD34⁺ hematopoietic stem cells [19], minimizing the risk of on-target off-tumor toxicity. In vitro and in vivo, IG9BBz CAR-T cells demonstrated potent cytotoxicity against IGSF9 positive AML cells. Although IG9BBz CAR-T cells exhibited comparable in vitro cytotoxicity to CD19-specific CAR-T cells (Supplementary Figure S5D), complete tumor remission was not achieved in the IGSF9⁺ AML xenograft model. Notably, residual tumor cells harvested 14 days post-treatment displayed markedly diminished IGSF9 surface expression (Supplementary Figure S6A-B), implicating antigen escape as a principal mechanism underlying the partial response. Recent studies reporting robust anti-AML efficacy with CAR-T cells directed against novel targets such as CD37, CD84, and CD96 have typically employed high effector doses (5–10 × 10⁶ cells per mouse) or low tumor burden models (e.g., 5 × 10³ MOLM13 cells per mouse); even under these optimized conditions, complete eradication remains challenging [24–26]. The incomplete tumor control observed here thus reflects the inherent resistance of AML cell lines to CAR-T therapy, which generally necessitates elevated effector-to-target ratios for durable clearance [27, 28]. This limitation potentially restricts the clinical applicability of IG9BBz CAR-T cells to patients with IGSF9-high disease. Future efforts to enhance therapeutic efficacy should focus on affinity maturation or epitope optimization of the anti-IGSF9 scFv to improve recognition of heterogeneous IGSF9 expression levels.

Nevertheless, this strategy leverages the dual role of IGSF9- its tumor-restricted expression and immunosuppressive function- enabling simultaneous clearance of malignant cells and disruption of the inhibitory niche. As the heterogeneous IGSF9 expression across the AML clone landscape could pose a significant barrier to durable remission in clinical settings, the potential emergence of tumor escape could be mitigated by employing tandem CAR constructs that co-target IGSF9 and AML-restricted antigens (e.g., CD33 or CD123) under OR-gate logic, thereby ensuring robust activity against both antigen-low subclones and microenvironmentally suppressive cells [29].

Limitations of this study include the use of immunodeficient AML models for CAR-T therapy, which cannot fully recapitulate endogenous T cell-AML interactions or allow assessment of graft-versus-host disease. Future studies will employ humanized models and primary AML xenografts with autologous CAR-T manufacturing to better evaluate patient-specific heterogeneity and immune dynamics. Moreover, this study does not assess potential on-target/off-tumor toxicity of IGSF9-directed therapies, which requires urgent investigation given its trace expression in neuronal tissues (Human Protein Atlas, proteinatlas.org) [30]. Currently, reliable preclinical models for evaluating CAR-T on-target/off-tumor toxicity remain limited. A human/mouse cross-reactive scFv would enable toxicity assessment in mice [31]; however, since our anti-IGSF9 scFv does not recognize mouse Igsf9, human IGSF9 transgenic mice are required for definitive toxicity evaluation. To mitigate on-target/off-tumor risk, several engineering strategies merit consideration: an AND-NOT gate design incorporating an inhibitory CAR (iCAR) to quench T-cell activity at nonmalignant tissue sites [32], or affinity-optimized scFv variants that spare normal cells expressing ultra-low IGSF9 levels while maintaining potent recognition of tumor-high expression [33].

## Conclusions

In summary, we delineate a cytokine-driven IGSF9-mediated resistance circuit that undermines CAR-T cell activity in AML and validate two complementary therapeutic strategies-IGSF9 blockade and IGSF9-directed CAR-T cells-to disrupt this immunosuppressive axis. These findings provide a mechanistic framework for next-generation immunotherapies that integrate tumor antigen targeting with reprogramming of the immune microenvironment, offering a promising path toward sustained remission in high-risk AML.

## Supplementary Information

Supplementary Material 1.

## Data Availability

All data generated or analyzed during this study are included in this published article and its supplementary information files.

## Abbreviations

AML: Acute myeloid leukemia
CAR: Chimeric antigen receptor
ECD: Extracellular domain
FBS: Fetal bovine serum
GVHD: Graft-versus-host disease
HR: Hazard ratio
IGSF9: Immunoglobulin superfamily member 9
KO: Knock out
LAG-3: Lymphocyte activation gene 3
MHC: Major histocompatibility complex
NSG mice: NOD/SCID-IL2Rg-null mice
PBMC: Peripheral blood mononuclear cells
PD-1: Programmed cell death protein 1
PD-L1: Programmed cell death protein ligand 1
ScFv: Single-chain variable fragment
STR: Short tandem repeats
TIM-3: T cell immunoglobulin and mucin domain-3
UnT: Untransduced T
WT: Wild type
