# Supplementary material for: Targeting immunoglobulin superfamily member 9 (IGSF9) to overcome acute myeloid leukemia resistance to CAR-T therapy
Source: J Exp Clin Cancer Res. 2026 May 19;45:157. doi: 10.1186/s13046-026-03740-4 (PMC13352843; doi:10.1186/s13046-026-03740-4)
Supplement: Supplementary file 1 — Supplementary Material 1. [file 13046_2026_3740_MOESM1_ESM.docx]

**Supplementary Figures**


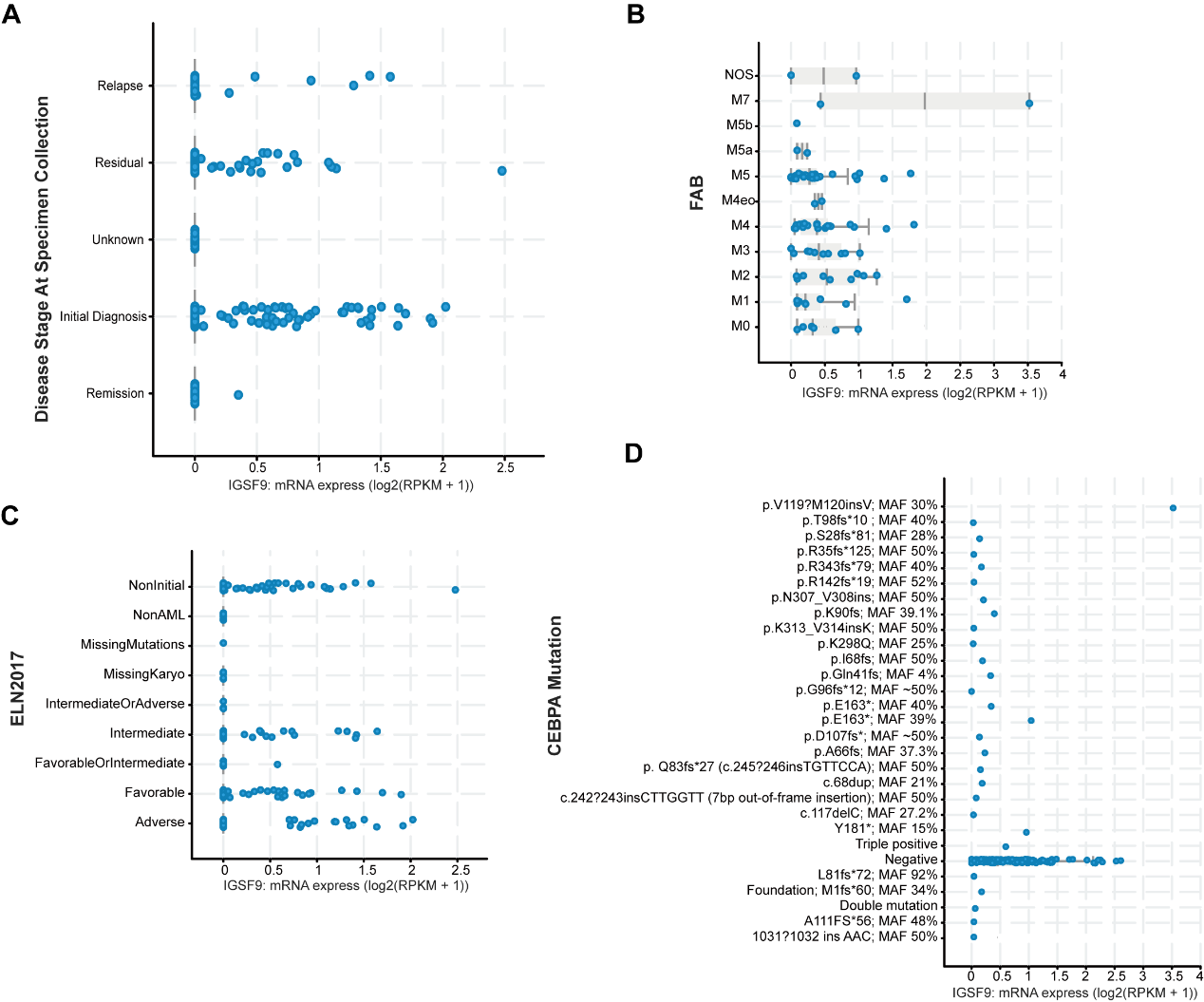


**Supplementary Figure S1. IGSF9 expression in AML patients with different disease stages (A), FAB types (B), ELN2017 characterizations (C) and CEBPA mutations** (Related to Figure 1).


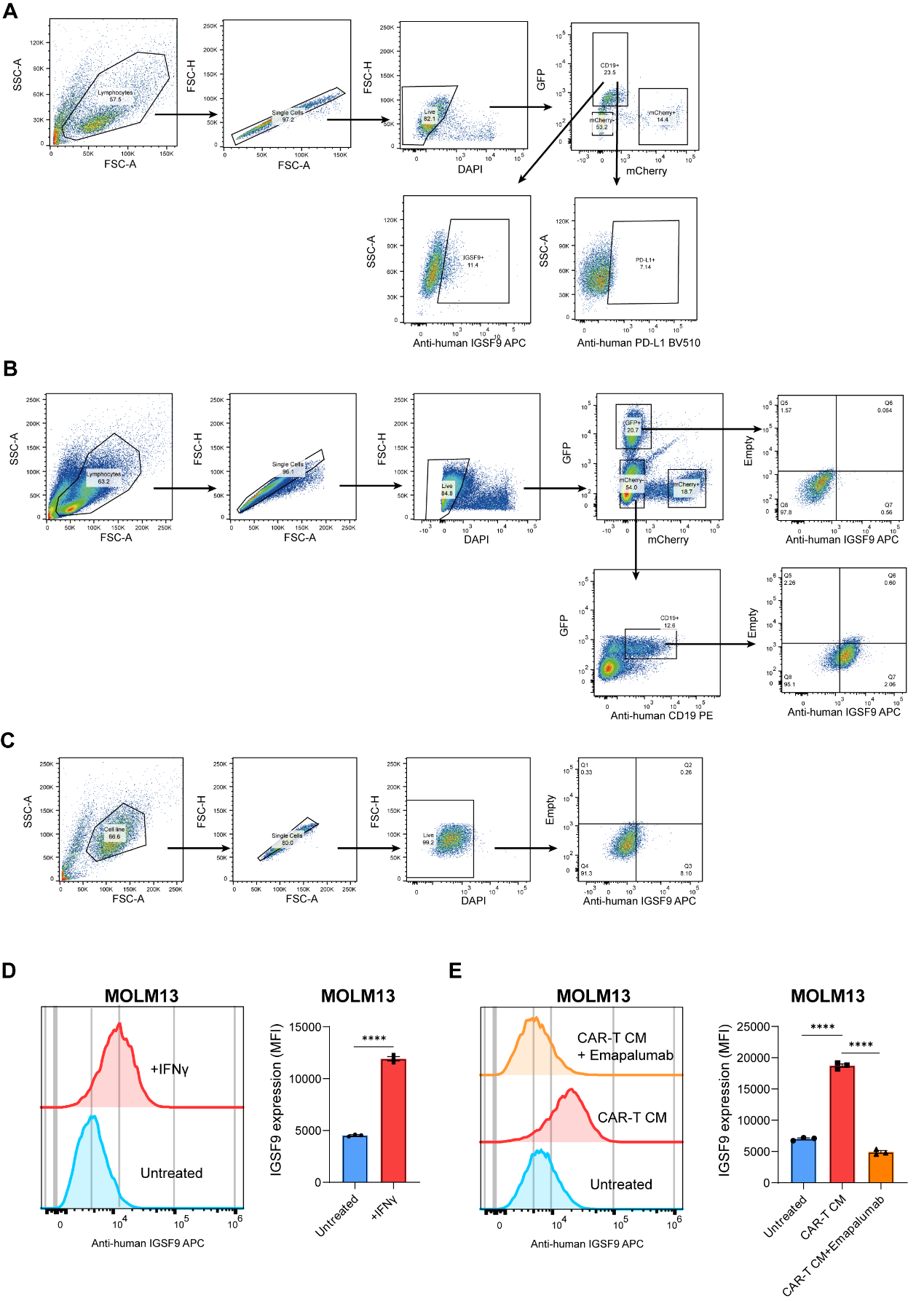


**Supplementary Figure S2.** **CAR-T cells derived IFNγ induced upregulation of IGSF9 in AML** (Related to Figure 2).
**(A-C)** Flow Cytometry Gating Strategies for In Vitro CAR-T/AML Co-cultures.

**(A)** Analysis of CAR-T cells co-cultured with MOLM13-CD19 cells.
**(B)** Analysis of CAR-T cells co-cultured with a MOLM13-GFP/MOLM13-CD19 cell mixture.
**(C)** Analysis of AML cells treated with conditioned medium from MOLM13/CAR-T co-cultures.

**(D)** IGSF9 upregulation in MOLM13 cells after treating with 100ng/ml IFNγ for 24 hours (mean ± SEM; n=3).

**(E)** IFNγ blocking abrogated CAR-T CM induced IGSF9 upregulation in AML. MOLM13 cells were treated with conditioned medium from MOLM13/33BBz CAR-T co-cultures (CAR-T CM) in the presence or absence of IFNγ blocking antibody (Emapalumab, 20 ng/mL). Surface IGSF9 expression was measured by flow cytometry versus untreated controls. (mean ± SEM; n=3).


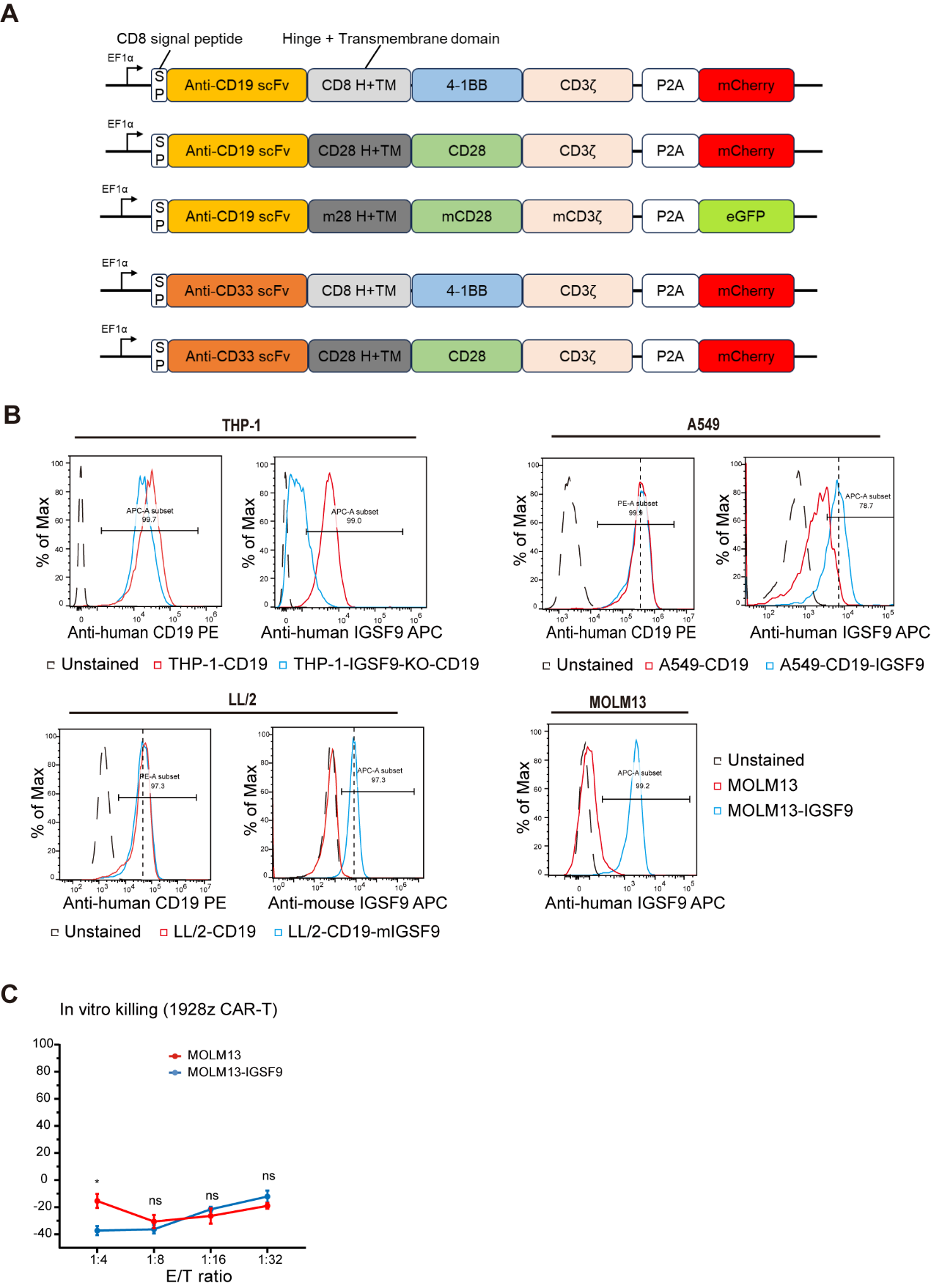


**Supplementary Figure S3. Target cells preparation and cytotoxicity assay** (Related to Figure 3).
**(A)** Schematic diagram of CAR constructs used in this experiment. Constructs consist of key domains including an anti-CD19 or CD33 scFv, hinge/transmembrane domain, and intracellular signaling domains comprising a co-stimulatory domain (4-1BB or CD28) and the CD3ζ domain. Each CAR was linked to a fluorescence protein (mCherry or eGFP) via a P2A sequence. Expression was driven by the EF1α promoter.

**(B)** Generation of CAR-T target cells with different IGSF9 expression. Surface expression of CD19 or IGSF9 on THP-1, A549, LL/2 and MOLM13 cells was verified by flow cytometry.

**(C)** Cytotoxicity of 1928z CAR-T cells against MOLM13 versus MOLM13 overexpressing IGSF9 (MOLM13-IGSF9).


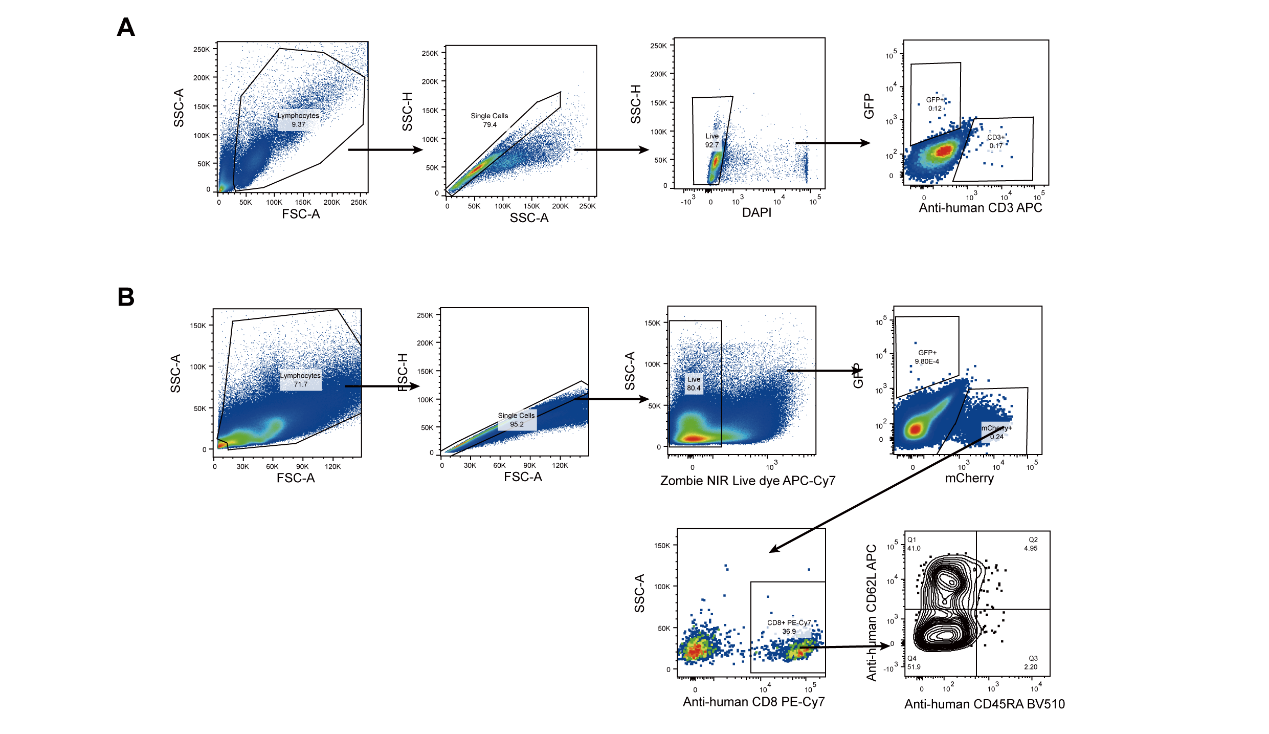


**Supplementary Figure S4. Flow Cytometry Gating Strategies for In Vivo CAR-T/Tumor Analysis** (Related to Figure 6).
**(A)** Analysis of peripheral blood CD3⁺ T cells and GFP⁺ tumor cells in NSG mice.
**(B)** Analysis of CAR-T cells (mCherry⁺) in spleen and bone marrow of NSG mice.


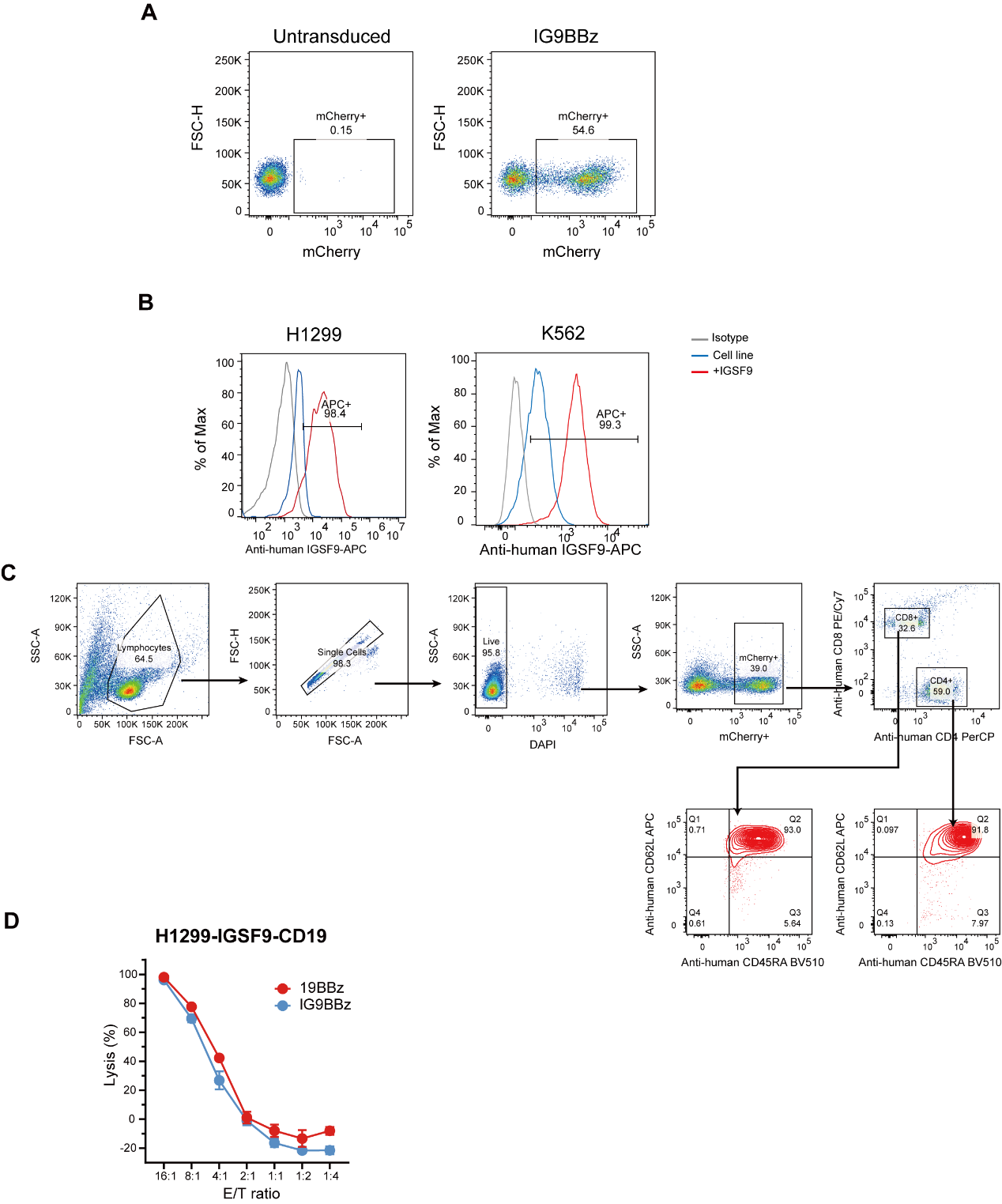


**Supplementary Figure S5. Validation of IGSF9-Specific CAR-T Function** (Related to Figure 7).
**(A)** Generation of IG9BBz CAR-T cells from human PBMCs.
**(B)** Generation of IGSF9-overexpressing H1299 and K562 cell lines.
**(C)** Differentiation analysis of CAR-T cells after co-culture with K562 or K562-IGSF9 cells.

**(D)** Compared cytotoxic effect of 19BBz and IG9BBz CAR-T cells towards IGSF9^+^CD19^+^ H1299 (H1299-IGSF9-CD19) cells (n=3).


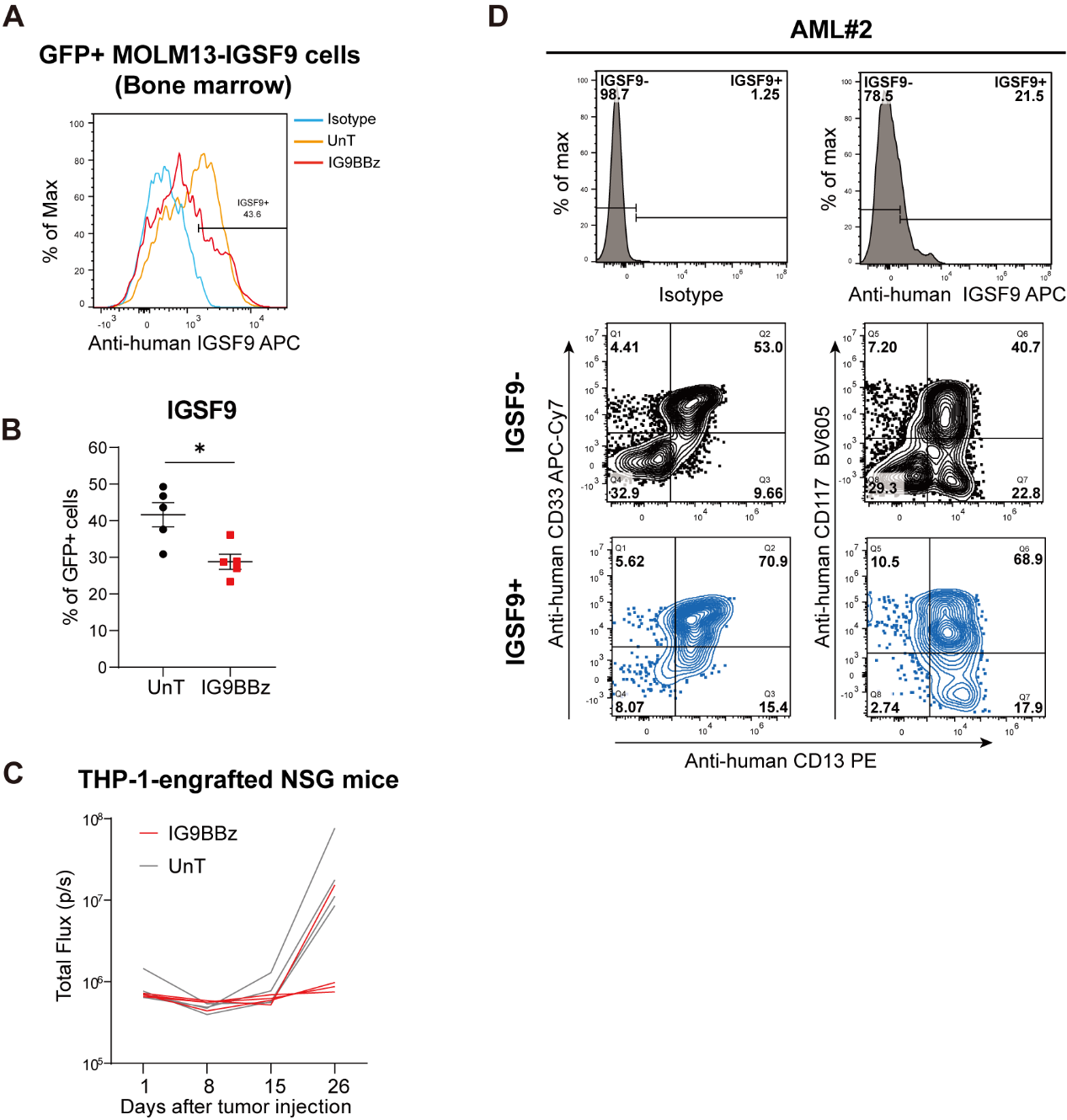


**Supplementary Figure S6. Validation of IGSF9-Specific CAR-T Function** (Related to Figure 8, 9).

**(A)** Representative flow cytometry of IGSF9 expression of the residual GFP^+^ tumor cells in bone marrow of mice.

**(B)** Quantification of IGSF9 expression from (A) (mean ± SEM; n=4 mice per group).

**(C)** Bioluminescence quantitative tumor burden of THP-1-engrafted NSG mice at serial timepoints. (total flux, p/s; mean ± SEM).

**(D)** AML-related markers expression of a patient-derive AML sample. The CD13, CD33 and CD117 expression of IGSF9^-^ and IGSF9^+^ populations were compared by flow cytometry.
